# Bereaved Next-of-Kin Narratives about Hospice Care in Assisted Living

**DOI:** 10.1093/geroni/igaf122.2603

**Published:** 2025-12-31

**Authors:** Nicole Dagen, Jacy Weems, Emily Gadbois, Melissa Clark, Grace Reed, Kali Thomas, Joan Teno, Emmanuelle Belanger

**Affiliations:** Brown University, Providence, Rhode Island, United States; Brown University School of Public Health, Providence, Rhode Island, United States; Brown University School of Public Health, Providence, Rhode Island, United States; Brown University, Providence, Rhode Island, United States; Brown University School of Public Health, Providence, Rhode Island, United States; Johns Hopkins University, Baltimore, Maryland, United States; Brown University, Providence, Rhode Island, United States; Brown University, Providence, Rhode Island, United States

## Abstract

There is limited evidence about the quality of end-of-life care provided in assisted living (AL), especially as related to various community-level care processes supporting dying in place. This study aimed to explore narratives of bereaved next-of-kin of deceased AL residents, focusing on end-of-life care quality and hospice utilization as a factor of AL retention policies for dying residents and staffing. We conducted 52 semi-structured telephone interviews with bereaved next-of-kin of persons who resided in AL during the last month of life across 38 states, purposefully oversampling racially and ethnically diverse ALs. We stratified qualitative data along care processes obtained through a nationally-representative survey of administrators at AL communities with 25 or more beds. Two coders applied exploratory thematic analysis to analyze narratives stratified by AL policy to retain residents in need of end-of-life care (vs. case-by-case vs. discharge) and any registered nurse (RN) on staff (yes/no). Next-of-kin cited the desire for more staff attention and avoiding late-life transitions as key motivating factors for enrollment in hospice. Even in AL communities with a policy to retain dying residents, participants reported receiving an ultimatum to either enroll their next-of-kin in hospice or move them to another setting like a nursing home. Regardless of RN availability, electing hospice enhanced staff access, particularly overnight presence and support for residents at risk of falling. Our study highlights the importance of hospice care in allowing dying residents to stay in place across AL communities with varying policies regarding end-of-life care and RN availability.

